# Stage III Lymph Node–Positive Non‐Small‐Cell Lung Cancer Patients With or Without Lymph Node Irradiation by Stereotactic Body Radiation Therapy: Propensity Score–Matched Analysis

**DOI:** 10.1111/crj.70191

**Published:** 2026-05-08

**Authors:** Zhen Jia, Fang Fang, Xiaofei Zhu, Yangsen Cao, Huojun Zhang

**Affiliations:** ^1^ Department of Radiation Oncology Changhai Hospital Affiliated to Navy Medical University Shanghai China

**Keywords:** chemotherapy, local control, lymph node‐positive, stage III NSCLC, stereotactic body radiation therapy

## Abstract

**Purpose:**

This study compared the local control (LC) and survival of patients with stage III lymph node–positive non‐small‐cell lung cancer (NSCLC) who received lymph node irradiation (LNs R+) and those who did not (LNs R−).

**Methods:**

We retrospectively reviewed patients with stage III LN–positive NSCLC who underwent stereotactic body radiotherapy (SBRT) with or without lymph node irradiation between January 2013 and December 2018. Using propensity score matching (PSM) analyses, we compared the rates of LC, progression‐free survival (PFS), overall survival (OS), and acute/late toxicities between the two groups.

**Results:**

We retrospectively analyzed 201 patients, of whom 52 received LN irradiation and 149 did not. The median LC and OS were 59.7 months (95% CI: 51.1–62.9 months) and 36.3 months (95% CI: 32.6–40.0 months), respectively. After the PSM analysis, 52 patients were included in each group. The median LC (30.6 months and not reached, HR 1.296, 95% CI: 0.733–2.294, *p* = 0.371) and OS (30.3 months and 31.6 months, HR 0.969, 95% CI: 0.628–1.496, *p* = 0.887) were similar between the LN R+ group and the LN R− group. Total tumor size, tumor location, and biologic equivalent dose (BED) were independently associated with LC (*p* < 0.05). The incidences of acute radiation esophagitis (*p* = 0.006) and late pulmonary interstitial fibrosis (*p* = 0.046) were significantly higher in the LN R+ group than in the LN R− group.

**Conclusions:**

SBRT is safe and effective in stage III LN–positive NSCLC patients. Omitting elective nodal irradiation achieves comparable survival to irradiating involved lymph nodes while significantly reducing toxicity. These findings support the development of novel combination strategies.

## Graphical abstract

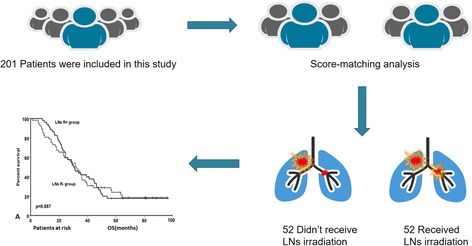

## 1 Introduction

Approximately 20% of patients with non‐small‐cell lung cancer (NSCLC) are initially diagnosed with unresectable stage III disease [1]. Prior to the PACIFIC trial, the standard treatment for such patients consisted of 6–7 weeks of thoracic radiotherapy combined with platinum‐based chemotherapy, which achieved a 5‐year overall survival (OS) rate of 10%–30% [2, 3]. Subsequently, the PACIFIC trial established the efficacy of durvalumab as consolidation therapy in patients with unresectable stage III NSCLC who had no disease progression following chemoradiotherapy (CRT). In parallel, the LAURA trial evaluated osimertinib as adjuvant therapy following definitive CRT in patients with unresectable stage III epidermal growth factor receptor (EGFR)–mutant NSCLC. Currently, consolidative immunotherapy after chemoradiation represents the standard of care for PDL1‐positive patients without disease progression, with a reported 3‐year OS of 57% [4]. Similarly, the LAURA regimen has demonstrated durable progression‐free survival (PFS) benefits and improved control of brain metastases in EGFR‐mutated patients [5]. Both studies utilized a treatment regimen of concurrent CRT delivered with conventional fractionation. Nevertheless, a subset of stage III NSCLC patients is ineligible for concurrent CRT due to factors such as advanced age, significant comorbidities, or patient refusal. For these individuals, alternative treatment approaches beyond conventional fractionation have attracted increasing clinical interest.

Over the past decades, advances in radiation technology enabled stereotactic body radiotherapy (SBRT) to deliver precisely targeted, ablative doses with a steep dose gradient, establishing this modality as a recommended treatment option for early‐stage and metastatic NSCLC [6, 7, 8, 9, 10]. Stage III NSCLC is typically characterized by the presence of a primary lung tumor accompanied by metastatic involvement of mediastinal lymph nodes (LNs) [11]. However, delivering high‐dose SBRT to involved LNs remained challenging due to their proximity to critical organs, including the esophagus, bronchi, major vessels, and heart [12]. Early clinical experience reported grade ≥ 3 toxicities in 46% of patients irradiated for central tumors or metastatic mediastinal LNs [13]. Several studies have investigated the integration of SBRT with conventional chemoradiation or its use as a single‐modality treatment in stage III disease, observing reduced local failure rates and improved clinical outcomes [11, 14, 15]. Anatomically, metastatic mediastinal LNs are often located closer to dose‐limiting organs at risk (OARs) than primary lung lesions, which further complicated dose escalation [16]. Consequently, subsequent research prioritized strategies that enabled safe delivery of ablative doses to primary tumors via novel radiotherapy techniques without increasing toxicity [16]. In an effort to improve the safety of SBRT, some protocols reduced initial target volumes or adopted more conservative fractionation schemes [11, 17]. Furthermore, in the case of patients with poor performance status, no survival benefits may be observed when both the primary tumor and metastatic LNs are irradiated, which may be ascribed to increased risk of adverse effects. Therefore, with the advances of systemic therapy, including immunotherapy and targeted therapy, reduced volume of stereotactic body radiation therapy with only involvement of the primary tumor may be an option.

Therefore, the purpose of this study was to compare the efficacy and safety between irradiated metastatic LNs (LNs R+) and nonirradiated metastatic LNs (LNs R−).

## 2 Methods

### 2.1 Patients

This retrospective study was approved by the Institutional Review Board of Changhai Hospital with a waiver of informed consent. Data of patients with NSCLC from 2013 to 2018 were extracted from the database. Stage III patients receiving SBRT were included in the study. All patients were staged according to the 8th edition of the American Joint Committee on Cancer (AJCC) TNM classification. Eligible patients were required to meet all of the following: (1) age ≥ 18 years with pathologically or cytologically confirmed NSCLC, (2) a Karnofsky Performance Status (KPS) score ≥ 70, and (3) the presence of LN metastasis (patients with T4N0 disease were excluded). LNs were considered clinically malignant if they measured ≥ 1 cm in the short‐axis diameter and exhibited definitive fluorodeoxyglucose (FDG) avidity. Pathological confirmation of LN metastases was pursued whenever feasible. Patients were excluded for any of the following: (1) a prior history of thoracic surgery or radiotherapy, (2) the presence of distant metastases (DM), or (3) severe comorbidities, active interstitial lung disease, or a synchronous second primary malignancy.

**FIGURE 1:**
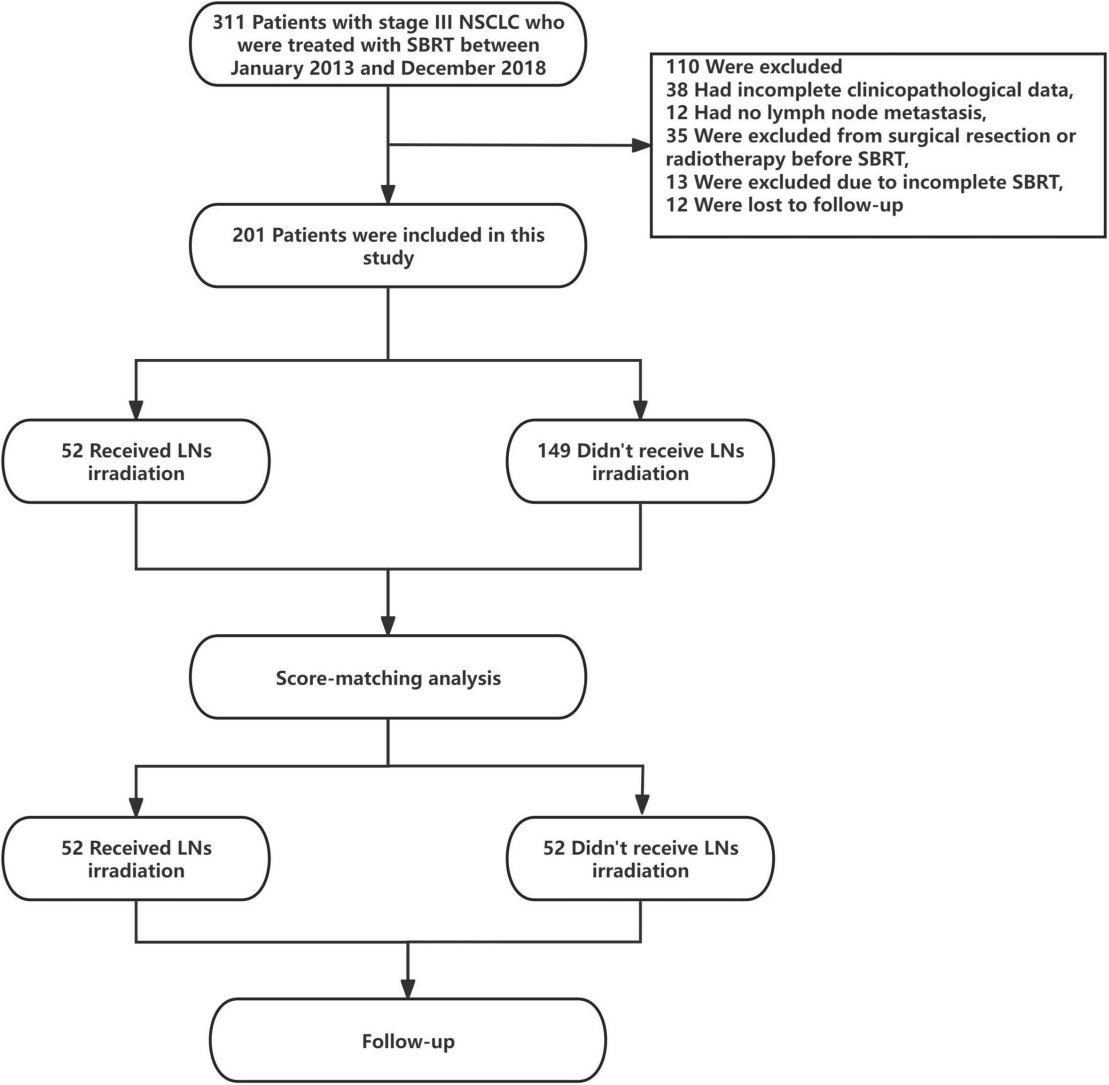
The flowchart.

Both induction and consolidation chemotherapy were allowed. According to the Radiation Therapy Oncology Group (RTOG) definition, primary central NSCLC was defined as lesions within 2 cm from the trachea, bronchial tree, esophagus, major vessels, heart, pericardium, or brachial plexus. We had de‐identified all patient details. The reporting of this study conformed to STROBE guidelines [18]. The flowchart was shown in Figure 1.

### 2.2 Radiation Therapy

SBRT was delivered via CyberKnife system (Accuray Incorporated, Sunnyvale, United States). Patients were immobilized in a vacuum bag in a supine position before simulations. All target volumes were delineated via computed tomography (CT) – based simulation images with at least 1.5 mm slice thickness. The acquired CT images were transferred to a three‐dimensional (3D) planning system (Accuray Incorporated, Sunnyvale, United States).

SBRT targets (the primary lesions and positive LNs) and doses would be initially determined based on the age, baseline comorbidities, TNM stage, and clinical imaging, prescribed by the treating radiation oncologist and/or based on discussion by a multidisciplinary team. According to the irradiation of the positive LNs, we divided the patients into two groups: the LN irradiation (LNs R+) group and the no LN irradiation (LNs R−) group.

The gross target volume (GTV) was defined as the primary visible tumor identified in the imaging examinations. The LN gross tumor volume (GTVnd) was defined as the mediastinal LNs with a ≥ 1‐cm diameter in the short axis. Multiple clusters of LNs on the CT images, areas of increased uptake on ^18^F‐ FDG‐PET, and pathologically confirmed LNs were also included in the GTVnd. In patients treated with induction chemotherapy, the post‐chemotherapy tumor volumes were defined as the GTV and GTVnd of which a short diameter was greater than 1 cm. LN with a short axis of < 1 cm after chemotherapy would no longer be irradiated.

The SBRT dose was 35–60 Gy administered in 5–10 fractions (F). GTV with the diameters of ≤ 3 cm surrounded by lung parenchyma would be given 50–60 Gy/5F. GTV with diameters > 3 cm or in contact with the chest wall would be delivered 35–60 Gy/6–7F. For GTV within 2 cm of the mediastinum or the brachial plexus, a dose of 35–60 Gy/5–10F was prescribed. The doses of GTVnd were determined by the radiation oncologists and could be consistent with or slightly lower than those of GTV.

The OARs such as the spinal cord, esophagus, heart, rest lung, trachea, proximal bronchial tree, and ipsilateral bronchus were referred to the American Association of Physicists in Medicine (AAPM) guidelines in TG‐101. The dosimetric parameters were extracted from dose‐volume histograms (DVHs) of the clinical plans' contours.

### 2.3 Systemic Therapy

Patients were allowed to receive a total of four cycles of chemotherapy, including induction chemotherapy and consolidation chemotherapy. Platinum‐based doublet chemotherapy, including carboplatin and cisplatin, was administered. The regimens used for squamous carcinoma and adenocarcinoma were mainly taxanes and pemetrexed, respectively. Chemotherapy schedules could be modified at physicians' discretion. Patients with extremely large tumors and a high risk of radiation‐related toxicities initially received induction chemotherapy to reduce tumor burdens. Given that the results of the PACIFIC trial were announced toward the end of our study period, patients were given the option to receive consolidative immunotherapy. For patients with driver gene‐positive mutations, targeted therapy was subsequently administered according to their specific mutation profile. For patients who were poor candidates for chemotherapy or declined chemotherapy, treatment with SBRT alone was recommended.

### 2.4 Statistics

To control the bias of critical clinicopathological factors between the LN R+ and LN R− groups, we utilized the propensity score matching (PSM) method [19]. In this manner, we generated 1:1 matched cohorts with a caliper width of 0.05. Initial clinicopathological and treatment characteristics were compared using the chi‐square test or Fisher's exact test and the nonparametric Wilcoxon rank sum test for univariate analysis. The absolute lymphocyte count (ALC) was also collected within 1 week before initiation of SBRT and 1 month after treatment. ΔALC was also calculated, which meant differences between pre‐ and post‐SBRT lymphocyte counts.

Locoregional control (LC), PFS, and OS were measured from the date of diagnosis. The Kaplan–Meier analysis was used to estimate LC, PFS, and OS. Cox proportional hazards regression was used for multivariate analysis. Factors with *p* < 0.10 in the univariable analysis were then included in the multivariable analysis.

Acute and late radiation–induced toxicities were evaluated based on the RTOG toxicity criteria [20]. We used logistic regression analysis to identify the prognostic factors of acute esophagitis and pulmonary interstitial fibrosis. All listed *p* values were two‐sided, and *p* < 0.05 was deemed significant. Statistical analyses were performed using SPSS version 25.0 (IBM Corporation, Armonk, NY, United States).

## 3 Results

### 3.1 Baseline Characteristics

We identified 311 patients with stage III NSCLC who were treated with SBRT between January 2013 and December 2018. Among these patients, 38 had incomplete clinicopathological data, 12 had no LN metastasis, 35 were excluded from surgical resection or radiotherapy before SBRT, 13 were excluded due to incomplete SBRT, and 12 were lost to follow‐up. Ultimately, 201 patients were included in this study, among which 52 received LN irradiation and 149 did not (Table 1).

**TABLE 1:** Baseline patient, treatment, and tumor characteristics of stage III NSCLC patients before and after PSM, *n* (%).

Variable	Before PSM				After PSM		
	All patient percentage (%)	LNs irradiation *N* = 52	LNs non‐irradiation *N* = 149	*p*	LN irradiation *N* = 52	LNs non‐irradiation N = 52	*p*
Age (yr)							
Md (range)	72 (38–89)	69.5 (45–86)	72 (38–89)	0.583	69.5 (45–86)	70.5 (45–88)	0.789
Gender							
Male	38 (18.9)	39 (75.0)	124 (83.2)	0.219	39 (75.0)	44 (84.6)	0.329
Female	163 (81.1)	13 (25.0)	25 (16.8)		13 (25.0)	8 (15.4)	
KPS							
90	68 (33.8)	19 (36.5)	49 (32.9)	0.626	19 (36.5)	14 (26.9)	0.452
80	118 (58.7)	28 (53.8)	90 (60.4)		28 (53.8)	35 (67.3)	
70	15 (7.5)	5 (9.6)	10 (6.7)		5 (9.6)	3 (5.8)	
History of smoking							
Yes	125 (62.2)	30 (57.7)	95 (63.8)	0.507	30 (57.7)	35 (67.3)	0.418
No	76 (37.8)	22 (42.3)	54 (36.2)		22 (42.3)	17 (32.7)	
Primary pulmonary diseases							
Yes	80 (39.8)	14 (26.9)	66 (44.3)	0.033	14 (26.9)	21 (40.4)	0.213
No	121 (60.2)	38 (73.1)	83 (55.7)		38 (73.1)	31 (59.6)	
T stage							
T1	36 (17.9)	9 (17.3)	27 (18.1)	0.952	9 (17.3)	4 (7.7)	0.042
T2	71 (35.3)	17 (32.7)	54 (36.2)		17 (32.7)	8 (15.4)	
T3	49 (24.4)	13 (25.0)	36 (24.2)		13 (25.0)	22 (42.3)	
T4	45 (22.4)	13 (25.0)	32 (21.5)		13 (25.0)	18 (34.6)	
N stage							
N1	22 (10.9)	4 (7.7)	18 (12.1)	0.071	4 (7.7)	13 (25.0)	0.042
N2	102 (50.7)	21 (40.4)	81 (54.4)		21 (40.4)	20 (38.5)	
N3	77 (38.3)	27 (51.9)	50 (33.6)		27 (51.9)	19 (36.5)	
TNM stage							
IIIa	96 (47.8)	17 (32.7)	79 (53.0)	0.037	17 (32.7)	17 (32.7)	0.772
IIIb	61 (30.3)	21 (40.4)	40 (26.8)		21 (40.4)	24 (46.2)	
IIIc	44 (21.9)	14 (26.9)	30 (20.1)		14 (26.9)	11 (21.2)	
Total tumor size (cm)							
Md (range)	4.2 (1.2–13.2)	5.5 (2.5–13.2)	4.0 (1.2–11.5)	0.176	5.5 (2.5–13.2)	5.5 (1.4–11.5)	0.813
Pathologic pattern							
Squamous cell carcinoma	88 (43.8)	26 (50.0)	62 (41.6)	0.656	26 (50.0)	28 (53.8)	0.565
Adenocarcinoma	103 (51.2)	24 (46.2)	79 (53.0)		24 (46.2)	20 (38.5)	
NOS	10 (5.0)	2 (3.8)	8 (5.4)		2 (3.8)	4 (7.7)	
Location							
Central	81 (40.3)	28 (53.8)	53 (35.6)	0.023	28 (53.8)	24 (46.2)	0.557
Peripheral	120 (59.7)	24 (46.2)	96 (64.4)		24 (46.2)	28 (53.8)	
BED_10_							
Md (range)	86.4 (52.7–132.0)	82.1 (52.7–69)	88.3 (56–132)	< 0.001	82.1 (52.7–132.0)	83.3 (59.5–132.0)	0.495
SUVmax							
Md (range)	11.5 (1.0–39.4)	12.2 (1.0–30.2)	11.4 (2.9–39.4)	0.480	12.2 (1.0–30.2)	14.4 (2.9–39.4)	0.646
ΔALC (K/mL)				0.002			0.022
Md (range)	0.6 (−1.2–7.6)	0.9 (−1.2–7.6)	0.5 (−1.2–7.6)		0.9 (−1.2–7.6)	0.5 (−0.7–7.6)	
Type of systemic therapy							
Induction CT + SBRT	105 (52.2)	20 (38.5)	85 (57.0)	0.030	20 (38.5)	30 (57.7)	0.194
Induction CT + SBRT + consolidation CT	19 (9.5)	10 (19.2)	9 (6.0)		10 (19.2)	4 (7.7)	
Induction CT + SBRT + consolidation CT + immunotherapy	4 (2.0)	1 (1.9)	3 (2.0)		1 (1.9)	1 (1.9)	
SBRT + consolidation CT	15 (7.5)	3 (5.8)	12 (8.1)		3 (5.8)	1 (1.9)	
SBRT alone	58 (28.9)	18 (34.6)	40 (26.8)		18 (34.6)	16 (30.8)	

Abbreviations: ALC, absolute lymphocyte count; BED, biologically effective dose; CT, chemotherapy; KPS, Karnofsky Performance Status; LNs, lymph nodes; NOS, non‐small‐cell lung cancer not otherwise specified; NSCLC, non‐small‐cell lung cancer; PSM, propensity score matching; SBRT, stereotactic body radiotherapy; SUV, standard uptake value.

The median follow‐up was 40.00 (range 5.28–100.70) months. The median LC was 59.7 months (95% CI: 51.1–62.9 months). The median OS and PFS were 36.3 months (95% CI: 32.6–40.0 months) and 16.0 months (95% CI: 14.6–17.4 months), respectively.

The median prescription dose of GTV in the LN R+ group and the LN R− group was 47.8 Gy (range: 35.0–60.0 Gy/5–8 f) and 48.0 Gy (range: 35.0–60.0 Gy/5–8 f) (*p* = 0.081). However, patients in the LN R+ group had lower BED_10_ of GTV than those in the LN R− group (median BED_10_: 82.1 Gy versus 88.3 Gy, *p* < 0.001). The median prescription dose of GTVnd in the LN R+ group was 42 Gy (range: 25.0–60.0 Gy/5–8 f). The median BED_10_ of GTVnd was 71.4 Gy (37.5–132.0 Gy). Among them, 25 patients (48.1%) had a lower dose of GTVnd than GTV (*p* < 0.001).

During the study period, only four patients (2%) underwent consolidation immunotherapy. In total, 63 patients (31.3%) received immune checkpoint inhibitors (ICIs) in the later‐line setting. Of the 201 patients enrolled, 33 (16.4%) harbored EGFR mutations, with no other actionable genomic alterations identified. After PSM, the overall proportion of EGFR mutations was 32.0%, comprising 10 patients in the LN R+ group and 23 patients in the LN R− group. After completion of study treatment, four patients in the LN R+ group and 10 patients in the LN R− group received osimertinib or other tyrosine kinase inhibitors (TKIs), respectively.

### 3.2 Clinical Characteristics and Survival of Propensity Score–Adjusted Patients

The baseline TNM stage and BED_10_ were used as independent variables for the PSM analysis. After propensity matching, there were 52 patients of each group included in the analysis (Table 1); there was no significant difference between the two groups in the case of total prescription doses and numbers of fractions. The ΔALC was higher in the LN R+ group than that in the LN R− group (*p* = 0.022). The doses of OARs were similar except that the esophagus mean dose and lung V5 in the LN R+ group were higher than those in the LN R− group (Table 2).

**TABLE 2:** Comparison of target coverage and normal tissue sparing with PSM groups.

Variable	LN irradiation median (range)	LN non‐irradiation median (range)	*p*
Total dose, Gy	47.8(35.0–60)	45.8 (35.0–60.0)	0.981
Number of fractions	6 (5–10)	6 (5–8)	0.111
BED_10_, Gy	82.1 (52.7–132.0)	83.3 (59.5–132.0)	0.495
Coverage(%)	88.1 (54.1–99.5)	90.6 (71.5–97.5)	0.056
PTV (*D*max), Gy	49.0 (40.6–56.8)	56.6 (35.2–72.3)	0.347
Spinal cord (*D*max), Gy	6.9 (1.4–17.3)	7.5 (1.8–18.8)	0.758
Spinal cord (*D*_0.35cm3_), Gy	10.2 (4.5–18.0)	11.9 (1.5–24.3)	0.711
Heart mean dose, Gy	5.3 (3.9–8.8)	3.8 (0.9–11.3)	0.499
Heart (*D*_15cm3_), Gy	13.4 (6.6–24.6)	15.3 (2.47–31.2)	0.060
Esophagus mean dose, Gy	6.9 (2.8–8.7)	4.0 (1.3–12.3)	0.041
Esophagus (*D*_5cm3_), Gy	10.8 (5.3–14.52)	12.7 (2.58–22.6)	0.427
Lung mean dose, Gy	7.8 (4.9–13.0)	6.1 (0.3–11.7)	0.239
Lung V5, %	52.4 (25.7–70.4)	25.4 (8.2–54.9)	0.005
Lung V20, %	4.9 (0.0–9.5)	4.6 (0.0–11.1)	0.530

Abbreviations: BED, biologically effective dose; *D*_0.35cm3_, *D*_15cm3_, *D*_5cm3_, dose received 0.35, 15, and 5 cm^3^ volume of organ at risk, respectively; *D*max, maximum point dose; LNs, lymph nodes; Lung V5, V20, volume of total lungs receiving 5Gy, 20 Gy, or more; PSM, propensity score matching; PTV, planning target volume.

**FIGURE 2:**
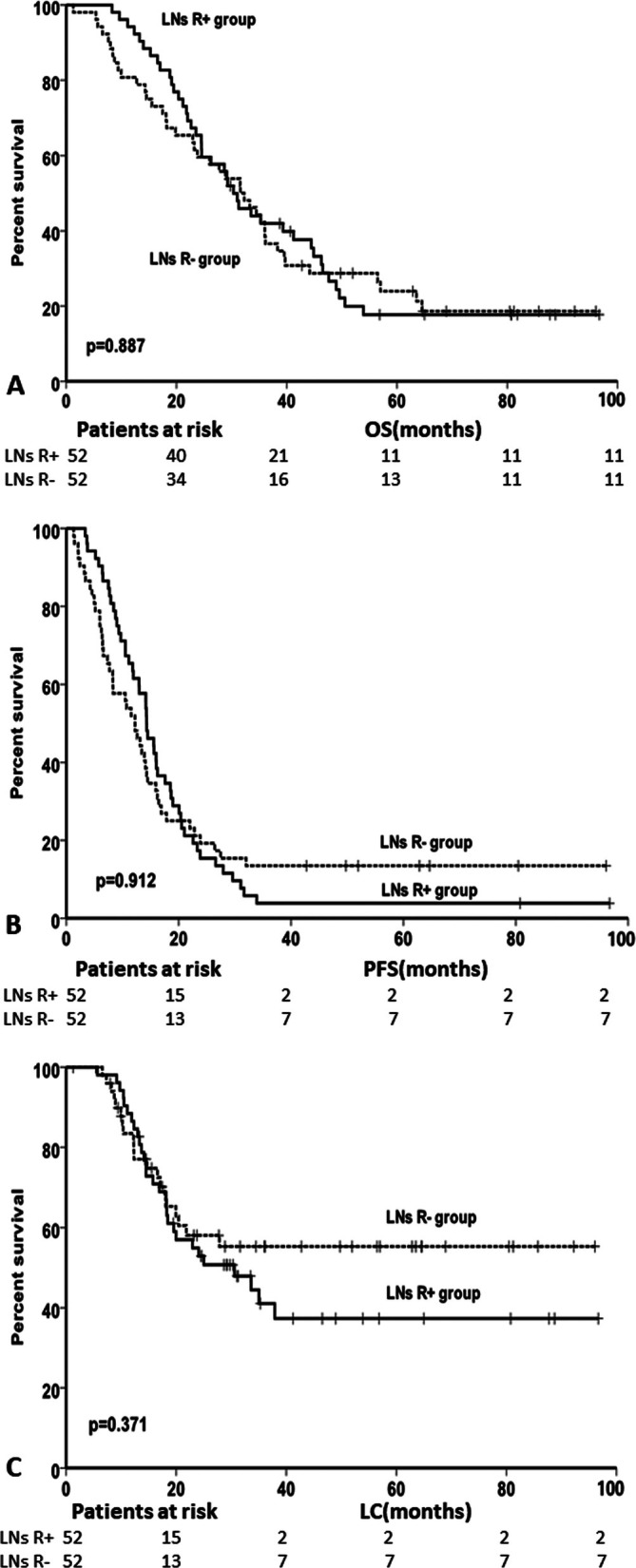
Survival curves for OS (A), PFS (B), and LC (C) stratified by with or without LN irradiation group. LC, local control; OS, overall survival; PFS, progression‐free survival.

The total median LC for the PSM groups was 33.6 months. The median LC was 30.6 months (95% CI: 15.2–46.0 months) and not reached for the LN R+ group and the LN R− group, respectively (*p* = 0.371) (Figure 2a). The estimated 1‐, 2‐, 3‐, and 5‐year LC rates for the LN R+ group were 86.5%, 54.9%, 41.0%, and 37.3%, respectively. The estimated 1‐, 2‐, 3‐, and 5‐year LC rates for the LN R− group were 83.5%, 58.0%, 55.3%, and 55.3%, respectively. Disease progression was recorded in 91.3% of patients (*n* = 95). The median PFS in the LN R+ group and the LN R− group were 14.3 months (95% CI: 12.6–15.9 months) and 12.2 months (95% CI: 9.1–15.3 months), respectively (*p* = 0.921) (Figure 2b). The estimated 1‐, 2‐, 3‐, and 5‐year PFS rates for the LN R+ group were 61.5%, 15.4%, 3.8%, and 3.8%, respectively. The estimated 1‐, 2‐, 3‐, and 5‐year PFS rates for the LN R− group were 51.9%, 19.2%, 13.5%, and 13.5%, respectively.

The median OS was 30.3 months (95% CI: 24.7–35.9 months) and 31.6 months (95% CI: 23.7–39.5 months) for the LN R+ group and the LN R− group, respectively (*p* = 0.887) (Figure 2c). The estimated 1‐, 2‐, 3‐, and 5‐year OS rates for the LN R+ group were 94.2%, 65.4%, 41.9%, and 17.7%, respectively. The estimated 1‐, 2‐, 3‐, and 5‐year OS rates for the LN R− group were 80.8%, 59.6%, 42.3%, and 23.9%, respectively.

The univariate and multivariate Cox regression analyses were shown in Table 3. Total tumor size, tumor location, and BED_10_ were independently associated with LC. The independent prognostic factor for PFS and OS was total tumor size. Overall, total tumor size was the only predictor of LC (*p* < 0.001), PFS (*p* = 0.007), and OS (*p* = 0.003). After propensity matching, 34.6% of patients subsequently received ICIs as part of their later‐line therapy. The median OS of the ICI group and the no ICI group were 35.3 months (95% CI: 13.3–57.3 months) and 30.3 months (95% CI: 26.7–33.9 months), respectively (*p* = 0.266).

**TABLE 3:** Prognostic factors for LC, PFS, and OS in PSM groups.

Locoregional control		Univariate			Multivariate		
Variables	Reference vs.	HR	95% CI	*p*	HR	95% CI	*p*
Lymph node irradiation	No vs. yes	1.296	0.733–2.294	0.373	NI		
Age (yr)	< 70 vs. ≥ 70	1.326	0.756–2.326	0.324	NI		
Sex	Female vs. male	1.289	0.643–2.588	0.474	NI		
History of smoking	No vs. yes	1.298	0.725–2.322	0.380	NI		
Primary pulmonary diseases	No vs. yes	0.875	0.476–1.607	0.666	NI		
TNM stage	IIIA vs. IIIB‐IIIC	1.539	0.815–2.906	0.183	NI		
T stage	T1–2 vs. T3–4	1.692	0.928–3.084	0.086	1.540	0.734–3.231	0.254
N stage	N1–2 vs. N3	1.192	0.681–2.089	0.538	NI		
Pathologic pattern	ADC vs. non‐ADC	1.635	0.918–2.910	0.095	1.375	0.755–2.505	0.297
Total tumor size (cm)	≤ 5.5 vs. > 5.5	2.638	1.484–4.689	0.001	3.345	1.797–6.225	< 0.001
Location	Central vs. peripheral	0.409	0.228–0.734	0.003	0.502	0.276–0.912	0.024
BED_10_ (Gy)	≤ 83.3 vs. > 83.3	0.096	0.038–0.243	< 0.001	0.081	0.031–0.210	< 0.001
ΔALC (K/mL)	≤ 0.6 vs. > 0.6	0.520	0.289–0.937	0.030	0.646	0.342–1.219	0.177

Progression‐free survival		Univariate			Multivariate		
Variables	Reference vs.	HR	95% CI	*p*	HR	95% CI	*p*
Lymph node irradiation	No vs. yes	0.980	0.653–1.470	0.921	NI		
Age (yr)	< 70 vs. ≥ 70	0.814	0.542–1.223	0.323	NI		
Sex	Demale vs. male	1.430	0.871–2.350	0.157	NI		
History of smoking	No vs. yes	1.261	0.832–1.913	0.274	NI		
Primary pulmonary diseases	No vs. yes	0.825	0.532–1.280	0.390	NI		
TNM stage	IIIA vs. IIIB‐IIIC	1.284	0.823–2.005	0.271	NI		
T stage	T1–2 vs. T3–4	1.522	1.001–2.315	0.049	1.341	0.570–3.156	0.501
N stage	N1–2 vs. N3	1.134	0.752–1.710	0.548	NI		
Pathologic pattern	ADC vs. non‐ADC	1.071	0.712–1.609	0.742	NI		
Total tumor size (cm)	≤ 5.5 vs. > 5.5	2.271	1.505–3.427	< 0.001	2.381	1.264–4.484	0.007
Location	Central vs. peripheral	0.662	0.441–0.994	0.047	0.657	0.359–1.202	0.173
BED_10_ (Gy)	≤ 83.3 vs. > 83.3	0.927	0.618–1.389	0.713	NI		
ΔALC (K/mL)	≤ 0.6 vs. > 0.6	1.012	0.676–1.516	0.954	NI		

Overall survival		Univariate			Multivariate		
Variables	Reference vs.	HR	95% CI	*p*	HR	95% CI	*p*
Lymph node irradiation	No vs. yes	0.969	0.628–1.496	0.887	NI		
Age (yr)	< 70 vs. ≥ 70	1.193	0.772–1.844	0.426	NI		
Sex	female vs. male	1.446	0.834–2.506	0.189	NI		
History of smoking	No vs. yes	1.133	0.724–1.771	0.585	NI		
Primary pulmonary diseases	No vs. yes	0.886	0.553–1.419	0.614	NI		
TNM stage	IIIA vs. IIIB‐IIIC	1.309	0.813–2.108	0.268	NI		
T stage	T1–2 vs. T3–4	1.964	1.229–3.139	0.005	1.379	0.529–3.595	0.510
N stage	N1–2 vs. N3	0.933	0.603–1.443	0.756	NI		
Pathologic pattern	ADC vs. non‐ADC	0.886	0.553–1.419	0.614	NI		
Total tumor size (cm)	≤ 5.5 vs. > 5.5	1.570	1.017–2.425	0.042	2.698	1.407–5.175	0.003
Location	Central vs. peripheral	0.524	0.338–0.813	0.004	0.648	0.337–1.246	0.193
BED_10_ (Gy)	≤ 83.3 vs. > 83.3	0.814	0.525–1.264	0.360	NI		
ΔALC (K/mL)	≤ 0.6 vs. > 0.6	0.900	0.583–1.389	0.633	NI		

Abbreviations: ALC, absolute lymphocyte count; BED, biologically effective dose; LC, local control; NI, not included in the multivariate model; OS, overall survival; PFS, progression‐free survival; PSM, propensity score matching.

### 3.3 Patterns of Local Failure of Propensity Score–Adjusted Patients

**FIGURE 3:**
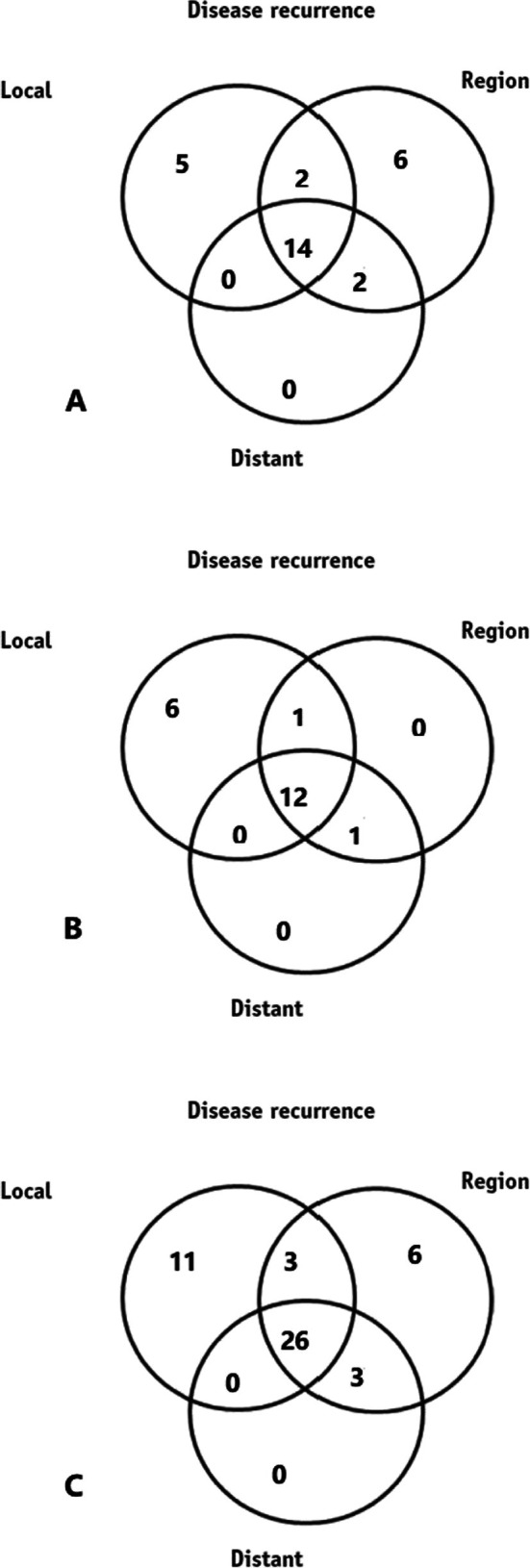
Locoregional failure patterns among (A) 29 patients with recurrent tumors in patients with LN irradiation; (B) 20 patients with recurrent tumors in patients with LN non‐irradiation; (C) 49 patients with recurrent tumors in the PSM cohort.

A total of 49 (47.1%) patients had local failure (Figure 3). Among these patients, 26 (53.1%) patients had in‐field and out‐field failure and DM; three (6.1%) patients had in‐field and out‐field failure; three (6.1%) patients developed out‐field failure and DM, while only out‐field and in‐field were observed in 6 (12.2%) and 11 (22.5%) patients. There was no statistical difference in the recurrence pattern between the two groups (*p* = 0.245).

In the LN non‐irradiation (LN R−) group, 35 patients (67.3%) received systematic treatment prior to SBRT. After systematic treatment, 65.7% (23/35) of patients had PR, 28.6% (10/35) had SD, and 5.7% (2/35) had PD, respectively. The reason for patients with PD was the enlargement of the primary lesion. After SBRT, 37.1% (13/35) of patients had locoregional progression, of which 12 patients experienced primary tumor enlargement and 10 patients had positive LN enlargement or the appearance of new positive LNs. Nine patients had both primary lesions enlargement and metastatic LNs, with only one patient experiencing regional LN metastases.

After PSM, 84 (80.8%) patients had a BED_10_ less than 100 Gy, in which 77 (91.7%) patients experienced disease progression. The rate of local progression and DM were 56.0% and 70.2%, respectively. However, in the BED_10_ ≥ 100 Gy group, only two patients (10%) experienced local progression.

### 3.4 Toxicity of Propensity Score–Adjusted Patients

The reported toxicities were summarized in Table 4. There were 18.3% patients (*n* = 19) with ≥ grade 3 acute toxicities in the PSM cohort. The acute radiation esophagitis was more frequent in the LN R+ group than in the LN R− group (32.7% vs. 11.5%, *p* = 0.006). In the LN R+ group, there were two patients with grade 3 acute esophagitis while no patients suffered this adverse event (*p* = 0.153). Grade 3 acute pneumonia was observed in eight (15.4%) and six (11.5%) patients in the LN R+ group and the LN R− group (*p* = 0.566). In the meanwhile, one patient died from radiation pneumonitis without evident disease progression 3 months after SBRT in the LN R− group. Additionally, 28 (26.9%) patients developed ≤ grade 2 late toxicities, whereas only one patient developed grade 3 late toxicities. The incidence of late pulmonary interstitial fibrosis (*p* = 0.046) was also significantly higher in the LN R+ group than in the LN R− group.

**TABLE 4:** Treatment‐related adverse events in PSM groups.

Variable	Grade	LNs irradiation *N* = 52 *N*, (%)	LNs non‐irradiation *N* = 52 *N*, (%)	*p*
Acute toxicity				
Hematological toxicities	Grade 0	45 (86.5)	46 (88.5)	0.102
	Grade 1	3 (5.8)	4 (7.7)	
	Grade 2	2 (3.8)	2 (3.8)	
	Grade 3	2 (3.8)	0 (0.0)	
Radiation esophagitis	Grade 0	35 (67.3)	46 (88.5)	0.006
	Grade 1	10 (19.2)	4 (7.7)	
	Grade 2	5 (9.6)	2 (3.8)	
	Grade 3	1 (1.9)	0 (0.0)	
	Grade 4	1 (1.9)	0 (0.0)	
Radiation pneumonia	Grade 0	36 (69.2)	41 (78.8)	0.177
	Grade 1	0 (0.0)	1 (1.9)	
	Grade 2	8 (15.4)	4 (7.7)	
	Grade 3	5 (9.6)	4 (7.7)	
	Grade 4	3 (5.8)	1 (1.9)	
	Grade 5	0 (0.0)	1 (1.9)	
Bronchopulmonary hemorrhage	Grade 0	50 (96.2)	52 (100.0)	0.180
	Grade 2	1 (1.9)	0 (0)	
	Grade 3	1 (1.9)	0 (0)	
Late toxicity				
Pulmonary interstitial fibrosis	Grade 0	39 (75.0)	45 (86.5)	0.046
	Grade 1	10 (19.2)	6 (11.5)	
	Grade 2	3 (5.8)	1 (1.9)	
Radiation esophagitis	Grade 0	47 (90.4)	48 (92.3)	0.180
	Grade 1	2 (3.8)	3 (5.8)	
	Grade 2	2 (3.8)	1 (1.9)	
	Grade 3	1 (1.9)	0 (0.0)	

Abbreviation: PSM, propensity score matching.

In further analysis, univariate analysis showed that LN irradiation (*p* = 0.009) and esophagus *D*_5cm3_ (*p* = 0.043) were associated with acute radiation esophagitis. The LN irradiation and age were independent influencing factors for radiation esophagitis and pulmonary interstitial fibrosis in multivariable analysis, respectively (Table 5). We also compared the relationship between the toxicity and survival outcomes, but there was no relationship between toxicity and LC, PFS, or OS in the two groups.

**TABLE 5:** Prognostic factors for acute esophagitis and pulmonary interstitial fibrosis in PSM groups.

Acute esophagitis		Univariate analysis			Multivariable analysis		
Variables	Reference vs.	OR	95% CI	*p*	OR	95% CI	*p*
Lymph node irradiation	No vs. yes	0.267	0.054–1.308	0.103	NI		
Age (yr)	< 70 vs. ≥ 70	1.111	0.242–5.107	0.892	NI		
Sex	Female vs. male	2.852	0.313–25.983	0.353	NI		
History of smoking	No vs. yes	1.852	0.387–8.871	0.441	NI		
Primary pulmonary diseases	No vs. yes	2.059	0.429–9.873	0.367	NI		
TNM stage	IIIA vs. IIIB‐IIIC	0.810	0.176–3.725	0.786	NI		
T stage	T1–2 vs. T3–4	7.000	0.784–62.525	0.082	1.573	0.095–26.002	0.837
N stage	N1–2 vs. N3	0.540	0.113–2.587	0.441	NI		
Total tumor size (cm)	≤ 5.5 vs. > 5.5	0.113	0.021–0.601	0.011	9.559	0.946–96.549	0.056
Location	Central vs. peripheral	2.172	0.235–29.097	0.494	NI		
BED_10_ (Gy)	≤ 83.3 vs. > 83.3	0.143	0.016–1.276	0.082	0.680	0.033–14.108	0.803
ΔALC (K/mL)	≤ 0.6 vs. > 0.6	0.706	0.136–3.658	0.678	NI		
PTV Dmax (Gy)	≤ 55.0 vs. > 55.0	6.300	0.705–56.287	0.099	1.128	0.068–18.632	0.933
Esophagus mean dose (Gy)	≤ 4.2 vs. > 4.2	3.706	0.661–20.765	0.136	NI		
Esophagus D_5cm3_ (Gy)	≤ 11.0 vs. > 11.0	9.625	1.075–86.175	0.043	9.939	0.542–182.296	0.122

Pulmonary interstitial fibrosis		Univariate analysis			Multivariable analysis		
Variables	Reference vs.	OR	95% CI	*p*	OR	95% CI	*p*
Lymph node irradiation	No vs. yes	0.519	0.120–2.233	0.378	NI		
Age (yr)	< 70 vs. ≥ 70	0.882	0.227–3.436	0.857	NI		
Sex	Female vs. male				NI		
History of smoking	No vs. yes	1.271	0.326–4.947	0.730	NI		
Primary pulmonary diseases	No vs. yes				NI		
TNM stage	IIIA vs. IIIB‐IIIC	0.625	0.160–2.441	0.499	NI		
T stage	T1–2 vs. T3–4	1.474	0.365–5.958	0.586	NI		
N stage	N1–2 vs. N3	0.481	0.119–1.945	0.305	NI		
Total tumor size (cm)	≤ 5.5 vs. > 5.5	2.286	0.519–10.057	0.274	NI		
Location	Central vs. peripheral	1.333	0.238–7.471	0.744	NI		
BED_10_ (Gy)	≤ 83.3 vs. > 83.3	0.679	0.168–2.743	0.586	NI		
ΔALC (K/mL)	≤ 0.6 vs. > 0.6	0.259	0.045–1.486	0.130			
PTV *D*max (Gy)	≤ 55.0 vs. > 55.0	2.246	0.509–9.907	0.285	NI		
Lung mean dose (Gy)	≤ 6.7 vs. > 6.7	1.271	0.326–4.947	0.730	NI		
Lung V5, %	≤ 39.8 vs. > 39.8	1.429	0.342–5.974	0.625	NI		
Lung V20, %	≤ 4.8 vs. > 4.8	2.083	0.516–8.407	0.302	NI		

Abbreviations: ALC, absolute lymphocyte count; BED, biologically effective dose; *D*_5cm3_, dose received 5 cm^3^ volume of organ at risk; *D*max, maximum point dose; PSM, propensity score matching; PTV, planning target volume.

## 4 Discussion

We retrospectively analyzed 201 patients with stage III LN–positive NSCLC. With a median follow‐up of 40 months, PSM analysis revealed that irradiation of metastatic LNs (LNs R+) did not confer additional benefits in LC, PFS, or OS compared with no irradiation of metastatic LNs (LNs R−); instead, it was associated with increased toxicity. To our knowledge, this is the first study to employ PSM analysis to compare outcomes of stage III NSCLC patients receiving LNs R+ with those receiving LNs R−.

In the case of recurrence patterns of stage III NSCLC, distant recurrence occurs in 50%–66% of cases and locoregional relapse occurred in 34%–50% [21], with in‐field failure at the primary site being the most common pattern of local relapse [22]. The most common toxicity is radiation‐induced esophagitis, which is closely associated with both dosimetric factors (i.e., the dose and volume of irradiated esophagus) and the use of concurrent chemotherapy [23, 24]. In light of these findings, priority should be given to delivering ablative doses to the primary tumor with accurate radiotherapy techniques, while minimizing the risk of toxicity. However, no consensus on the optimal target volume delineation for SBRT in node‐positive stage III NSCLC has yet been reached.

Recent studies have demonstrated the efficacy of SBRT for stage III NSCLC patients. In Cong et al.’s study, both the primary tumor and LNs were irradiated with a moderate median BED_10_ of 59.5 Gy. The LC rate was 61%, and the incidence of grade ≥ 3 toxicities was 9.8% [17]. In another study, SBRT was delivered for a dose boost following conventional CRT, achieving safe and effective local control in 37 patients with inoperable stage II‐III NSCLC. The SBRT boost was delivered to the residual disease (excluding LNs) 1 month after completion of CRT [25]. These divergent strategies highlight the ongoing uncertainty regarding optimal target volume selection.

One concern regarding SBRT only for primary tumors without metastatic LNs in these patients is the potential risk of locoregional relapse. However, previous studies have demonstrated that the role of radiotherapy in locally advanced NSCLC has shifted from a purely locoregional treatment to an immunomodulatory agent that synergizes with the immune system and reshapes the tumor microenvironment [26, 27]. Integration of SBRT with systemic therapy represents a mainstream strategy to improve therapeutic efficacy [28]. Emerging evidence from the neoadjuvant setting provides strong support for this paradigm shift. In the randomized, controlled Phase 2 trial by Altorki et al. [29], SBRT to the primary tumor followed by neoadjuvant immunotherapy yielded a major pathological response (MPR) rate of 53%, half of which were pathological complete responses (pCR) with acceptable safety. Among patients with stage IIIA disease, the MPR rate was 33.3%, and the pCR rate was 25.0%. In the study by Zhao et al. [28], SBRT achieved an MPR rate of 76% in patients with IIB‐IIIA stage. These encouraging neoadjuvant data demonstrated that SBRT to the primary tumor alone, combined with effective systemic therapy, can achieve high pathologic response rates without irradiating involved LNs. In our cohort, 49 (41.7%) patients developed local failure, of whom 26 (53.1%) also had concurrent DM. Out‐of‐field failure occurred in only 6 (12.2%) patients. The local failure rate was acceptably low in node‐positive stage III NSCLC, suggesting that omitting LN irradiation did not compromise LC. Notably, a large proportion of patients experienced both local failure and DM. This suggests that more intensive adjuvant systemic regimens and active surveillance should be prioritized over sole local irradiation of metastatic LNs. Our study contributes to this evolving paradigm by exploring optimal target volume delineation for SBRT in stage III NSCLC.

Importantly, we analyzed the incidence of local failure and identified factors associated with LC. Multivariate Cox regression analysis revealed that total tumor size, tumor location, and BED_10_ were independently associated with LC. Specifically, large tumor size was the only independent prognostic factor of poorer LC, PFS, and OS. These findings underscored the importance of patient selection and sufficient dose delivery to optimize outcomes.

Beyond failure patterns and prognostic factors, potential immune biomarkers were investigated. First, omitting LN irradiation may preserve antitumor immunity. We found that metastatic LN irradiation significantly reduced the ALC, a marker of post‐radiotherapy immune status [30] and prognostic factor in NSCLC [31, 32]. This aligned with preclinical data from Marciscano et al., who demonstrated that irradiation of tumor‐draining LNs decreased tumor‐infiltrating immune cells, suppressed chemokine expressions, and impaired survival when combined with immunotherapy [27]. These findings highlighted the importance of taking immunomodulatory effects of SBRT into target volume delineations. Consistent with this, our results suggested that unnecessary nodal irradiation may weaken the abscopal effect potentially triggered by high‐dose SBRT [33]. The absence of significant differences in recurrence patterns or OS between groups supported that SBRT alone may stimulate systemic immune effects, which may be compromised by elective nodal irradiation (ENI).

In parallel with immune considerations, our data emphasized the paramount importance of adequate dose delivery to the primary tumor. The local progression rate was 56.0% in the BED_10_ < 100 Gy group and 10.0% in the BED_10_ ≥ 100 Gy group. This suggested that for locally advanced NSCLC, BED_10_ ≥ 100 Gy to the primary tumor was crucial for achieving optimal LC. Similarly, many studies [34, 35] have shown that BED_10_ ≥ 100 Gy confers survival benefits in stage III NSCLC. In the Phase II study by Heinzelling et al. [34], all patients received SBRT (BED_10_ ≥ 100 Gy) to the primary tumor followed by conventionally fractionated chemoradiation to 60 Gy in 2 Gy fractions to the involved LNs. The median OS was 40.8 months, with DM being the main reason for failure in 26% of patients. Notably, only 2% of patients experienced local failure after SBRT and 6% experienced regional recurrence. These findings collectively stressed the importance of delivering ablative doses to the primary tumor.

Having established the efficacy of primary tumor‐focused SBRT, we also evaluated its impact on treatment‐related toxicity. Radiation‐induced acute and late toxicities are critically dependent on the volume of irradiated normal tissue, particularly in stage III NSCLC patients who commonly receive concurrent systemic therapy [36]. Severe (≥ Grade 3) esophageal and pulmonary toxicities remain the primary dose‐limiting concerns. In this context, minimizing the irradiated volume offers a direct strategy to improve the therapeutic index. Our study provides clear dosimetric and clinical evidence supporting this approach. The LN R+ group exhibited significantly higher lung V5 and mean esophageal dose, which directly translated into increased risk of toxicity. Patients receiving metastatic LN irradiation experienced significantly higher rates of acute radiation esophagitis and late pulmonary interstitial fibrosis. Multivariate analysis confirmed LN irradiation as an independent risk factor for radiation‐induced esophagitis. Although we observed no direct relationship between these toxicities and OS in this cohort, smaller mediastinal radiation fields would substantially decrease radiation exposure of adjacent OARs, thereby lowering the incidence of adverse effects. Importantly, this toxicity mitigation strategy may also create opportunities for safe dose escalation to the primary tumor, potentially further improving LC. These findings aligned with historical comparisons between ENI and involved‐field radiotherapy (IFRT). Studies from the pre‐immunotherapy era consistently demonstrated that ENI increased toxicity without conferring a clear survival benefit compared with more limited IFRT [36, 37, 38]. More recent prospective studies of hypofractionated regimens continued to report considerable toxicity, with grade 3 or greater pneumonitis ranging from 9.5% to 14% and grade 3 esophagitis from 2% to 5.3% [39]. These results reinforced that careful patient selection and target volume reduction can meaningfully improve the therapeutic ratio in stage III NSCLC, enhancing treatment tolerability without compromising oncologic efficacy.

Our study has several limitations. First, as this was a retrospective study, some patients did not receive uniform systemic treatment, which may have affected survival outcomes. Second, following PSM analysis, the sample size in each group was relatively small. Some baseline clinical pathological factors were different between groups, which may also have influenced our results. Finally, most patients were treated before the publication of the PACIFIC trial and LAURA trials [4, 5] and did not receive maintenance therapy. Future prospective studies may further reveal more surprising results regarding the combination of SBRT with immunotherapy that exempts LNs irradiation.

## 5 Conclusions

Our study demonstrates that SBRT is safe and effective for selected patients with stage III LN–positive NSCLC. Omitting metastatic LN irradiation yields survival outcomes comparable with nodal irradiation, with the critical benefits of reduced acute esophagitis, lower late pulmonary fibrosis, and preserved ALC—a potential biomarker that may indicate the improved efficacy of immunotherapy. These findings challenge the routine practice of nodal irradiation in the SBRT era and support primary tumor‐focused dose escalation with reduced target volumes, providing a rationale for future combination strategies with immunotherapy.

## Author Contributions

All authors contributed to the study's conception and design. Material preparation, data collection, and analysis were performed by F.F., Z.J., X.Z., Y.S.C., and H.Z. The first draft of the manuscript was written by Z.J., and all authors commented on previous versions of the manuscript. All authors contributed to the article and approved the submitted version.

## Funding

This work was supported by the Shanghai Health Commission Leading Talent Program (2022LJ019), and Changhai Hospital, Navy Medical University [2020YPT004].

## Ethics Statement

This study was performed in accordance with the Declaration of Helsinki. This human study was approved by Ethics Committee of Medical Faculty, Changhai Hospital Affiliated to Navy Medical University—approval: CHEC2024‐140. The study was not registered as a clinical trial because this study was a retrospective analysis of hospital medical records. Adult participant consent was not required because the data were only analyzed in an anonymized form.

## Conflicts of Interest

The authors declare no conflicts of interest.

## Data Availability

The data presented in this study are available on request from the corresponding author in an anonymized form after data privacy check. The data are not publicly available due to data privacy regulations.

## References

[1] P. K. Cheema , J. Rothenstein , B. Melosky , A. Brade , and V. Hirsh , “Perspectives on Treatment Advances for Stage III Locally Advanced Unresectable Non‐Small‐Cell Lung Cancer,” Current Oncology 26 (2019): 37–42, 10.3747/co.25.4096. 30853796 PMC6380636

[2] J. D. Bradley , R. Paulus , R. Komaki , et al., “Standard‐Dose Versus High‐Dose Conformal Radiotherapy With Concurrent and Consolidation Carboplatin Plus Paclitaxel With or Without Cetuximab for Patients With Stage IIIA or IIIB Non‐Small‐Cell Lung Cancer (RTOG 0617): a Randomised, Two‐by‐Two Factorial Phase 3 Study,” Lancet Oncology 16 (2015): 187–199, 10.1016/S1470-2045(14)71207-0. 25601342 PMC4419359

[3] W. J. Curran, Jr. , R. Paulus , C. J. Langer , et al., “Sequential Vs. Concurrent Chemoradiation for Stage III Non‐Small Cell Lung Cancer: Randomized Phase III Trial RTOG 9410,” Journal of the National Cancer Institute 103, no. 19 (2011): 1452–1460, 10.1093/jnci/djr325. 21903745 PMC3186782

[4] D. R. Spigel , C. Faivre‐Finn , J. E. Gray , et al., “Five‐Year Survival Outcomes From the PACIFIC Trial: Durvalumab After Chemoradiotherapy in Stage III Non–Small‐Cell Lung Cancer,” Journal of Clinical Oncology 40 (2022): 1301–1311, 10.1200/JCO.21.01308. 35108059 PMC9015199

[5] S. Lu , T. Kato , X. Dong , et al., “Osimertinib After Chemoradiotherapy in Stage III EGFR‐Mutated NSCLC,” New England Journal of Medicine 391, no. 7 (2024): 585–597, 10.1056/NEJMoa2402614. 38828946

[6] C. M. Bestvina , K. B. Pointer , T. Karrison , et al., “A Phase 1 Trial of Concurrent or Sequential Ipilimumab, Nivolumab, and Stereotactic Body Radiotherapy in Patients With Stage IV NSCLC Study,” Journal of Thoracic Oncology 17 (2022): 130–140, 10.1016/j.jtho.2021.08.019. 34500113

[7] D. J. Tandberg , B. C. Tong , B. G. Ackerson , and C. R. Kelsey , “Surgery Versus Stereotactic Body Radiation Therapy for Stage I Non‐Small Cell Lung Cancer: a Comprehensive Review,” Cancer 124 (2018): 667–678, 10.1002/cncr.31196. 29266226

[8] S. S. Lo , A. J. Fakiris , E. L. Chang , et al., “Stereotactic Body Radiation Therapy: a Novel Treatment Modality,” Nature Reviews. Clinical Oncology 7 (2010): 44–54, 10.1038/nrclinonc.2009.188. 19997074

[9] P. Iyengar , B. D. Kavanagh , Z. Wardak , et al., “Phase II Trial of Stereotactic Body Radiation Therapy Combined With Erlotinib for Patients With Limited But Progressive Metastatic Non‐Small‐Cell Lung Cancer,” Journal of Clinical Oncology 32, no. 34 (2014): 3824–3830, 10.1200/JCO.2014.56.7412. 25349291

[10] R. D. Timmerman , R. Paulus , H. I. Pass , et al., “Stereotactic Body Radiation Therapy for Operable Early‐Stage Lung Cancer: Findings From the NRG Oncology RTOG 0618 Trial,” JAMA Oncology 4 (2018): 1263–1266, 10.1001/jamaoncol.2018.1251. 29852037 PMC6117102

[11] O. L. Alcibar , E. Nadal , I. Romero Palomar , and A. Navarro‐Martin , “Systematic Review of Stereotactic Body Radiotherapy in Stage III Non‐Small Cell Lung Cancer,” Translational Lung Cancer Research 10 (2021): 529–538, 10.21037/tlcr-2020-nsclc-04. 33569334 PMC7867744

[12] S. Cozzi , E. Alì , L. Bardoscia , et al., “Stereotactic Body Radiation Therapy (SBRT) for Oligorecurrent/Oligoprogressive Mediastinal and Hilar Lymph Node Metastasis: a Systematic Review,” Cancers 14 (2022): 2680, 10.3390/cancers14112680. 35681659 PMC9179886

[13] R. Timmerman , R. McGarry , C. Yiannoutsos , et al., “Excessive Toxicity When Treating Central Tumors in a Phase II Study of Stereotactic Body Radiation Therapy for Medically Inoperable Early‐Stage Lung Cancer,” Journal of Clinical Oncology 24 (2006): 4833–4839, 10.1200/JCO.2006.07.5937. 17050868

[14] F. Arcidiacono , P. Anselmo , M. Casale , et al., “STereotactic Ablative RadioTherapy in NEWly Diagnosed and Recurrent Locally Advanced Non‐Small Cell Lung Cancer Patients Unfit for ConcurrEnt RAdio‐Chemotherapy: Early Analysis of the START‐NEW‐ERA Non‐Randomised Phase II Trial,” International Journal of Radiation Oncology, Biology, Physics 115, no. 4 (2023): 886–896, 10.1016/j.ijrobp.2022.10.025. 36288758

[15] Z. Jia , F. Fang , Y. Cao , et al., “Efficacy and Toxicity of Stereotactic Body Radiotherapy for Un‐Resectable Stage III Non‐Small Cell Lung Cancer Patients Unfit for Concurrent Chemoradiation Therapy: a Retrospective Study,” Radiation Oncology 18, no. 1 (2023): 140, 10.1186/s13014-023-02333-1. Erratum in: Radiation Oncology 2023;18(1):169. 37620952 PMC10463766

[16] D. Caivano , P. Bonome , D. Pezzulla , et al., “Stereotactic Body Radiation Therapy for the Treatment of Lymph Node Metastases: a Retrospective Mono‐Institutional Study in a Large Cohort of Patients,” Frontiers in Oncology 13 (2023): 1163213, 10.3389/fonc.2023.1163213. 37601675 PMC10435736

[17] Y. Cong , B. Sun , J. Wang , et al., “Outcomes and Toxicity of Stereotactic Body Radiation Therapy for Advanced Stage Ultra‐Central Non‐Small Cell Lung Cancer,” Thoracic Cancer 10 (2019): 1567–1575, 10.1111/1759-7714.13105. 31187604 PMC6610283

[18] E. von Elm , D. G. Altman , M. Egger , et al., “The Strengthening the Reporting of Observational Studies in Epidemiology (STROBE) Statement: Guidelines for Reporting Observational Studies,” Journal of Clinical Epidemiology 61, no. 4 (2008): 344–349, 10.1016/j.jclinepi.2007.11.008. 18313558

[19] R. B. D'Agostino , “Propensity Score Methods for Bias Reduction in the Comparison of a Treatment to a Non‐Randomized Control Group,” Statistics in Medicine 17 (1998): 2265–2281, 10.1002/(sici)1097-0258(19981015)17:19<2265::aid-sim918>3.0.co;2-b. 9802183

[20] J. D. Cox , J. Stetz , and T. F. Pajak , “Toxicity Criteria of the Radiation Therapy Oncology Group (RTOG) and the European Organization for Research and Treatment of Cancer (EORTC),” International Journal of Radiation Oncology, Biology, Physics 31 (1995): 1341–1346, 10.1016/0360-3016(95)00060-C. 7713792

[21] Expert Panel on Thoracic Imaging , R. Madan , R. H. El Alam , et al., “ACR Appropriateness Criteria Lung Cancer‐Surveillance After Therapy,” Journal of the American College of Radiology 22, no. 5S (2025): S319–S342, 10.1016/j.jacr.2025.02.025. 40409885

[22] S. Garg , B. T. Gielda , K. Kiel , et al., “Patterns of Locoregional Failure in Stage III Non‐Small Cell Lung Cancer Treated With Definitive Chemoradiation Therapy,” Practical Radiation Oncology 4, no. 5 (2014): 342–348, 10.1016/j.prro.2013.12.002. 25194104

[23] D. J. Herr , H. Yin , D. Bergsma , et al., “Factors Associated With Acute Esophagitis During Radiation Therapy for Lung Cancer,” Radiotherapy and Oncology 197 (2024): 110349, 10.1016/j.radonc.2024.110349. 38815695

[24] A. M. Brade , H. Bahig , A. Bezjak , et al., “Esophagitis and Pneumonitis Related to Concurrent Chemoradiation ± Durvalumab Consolidation in Unresectable Stage III Non‐Small‐Cell Lung Cancer: Risk Assessment and Management Recommendations Based on a Modified Delphi Process,” Current Oncology 31, no. 11 (2024): 6512–6535, 10.3390/curroncol31110483. 39590114 PMC11593044

[25] S. Kumar , J. Feddock , X. Li , et al., “Update of a Prospective Study of Stereotactic Body Radiation Therapy for Post‐Chemoradiation Residual Disease in Stage II/III Non‐Small Cell Lung Cancer,” International Journal of Radiation Oncology*Biology*Physics 99 (2017): 652–659, 10.1016/j.ijrobp.2017.07.036. 29280459

[26] J. Huang , W. S. M. E. Theelen , Z. Belcaid , et al., “Combination of Pembrolizumab and Radiotherapy Induces Systemic Antitumor Immune Responses in Immunologically Cold Non‐Small Cell Lung Cancer,” Nature Cancer 6, no. 10 (2025): 1676–1692, 10.1038/s43018-025-01018-w. 40696153 PMC12559004

[27] A. E. Marciscano , A. Ghasemzadeh , T. R. Nirschl , et al., “Elective Nodal Irradiation Attenuates the Combinatorial Efficacy of Stereotactic Radiation Therapy and Immunotherapy,” Clinical Cancer Research 24 (2018): 5058–5071, 10.1158/1078-0432.CCR-17-3427. 29898992 PMC6532976

[28] Z. R. Zhao , S. L. Liu , T. Zhou , et al., “Stereotactic Body Radiotherapy With Sequential Tislelizumab and Chemotherapy as Neoadjuvant Therapy in Patients With Resectable Non‐Small‐Cell Lung Cancer in China (SACTION01): a Single‐Arm, Single‐Centre, Phase 2 Trial,” Lancet Respiratory Medicine 12, no. 12 (2024): 988–996, 10.1016/S2213-2600(24)00215-7. 39305910

[29] N. K. Altorki , T. E. McGraw , A. C. Borczuk , et al., “Neoadjuvant Durvalumab With or Without Stereotactic Body Radiotherapy in Patients With Early‐Stage non‐Small‐Cell Lung Cancer: a Single‐Centre, Randomised Phase 2 Trial,” Lancet Oncology 22, no. 6 (2021): 824–835, 10.1016/S1470-2045(21)00149-2. 34015311

[30] S. Takanen , M. Bottero , P. Nisticò , and G. Sanguineti , “A Systematic Review on the Impact of Hypofractionated and Stereotactic Radiotherapy on Immune Cell Subpopulations in Cancer Patients,” Cancers 14 (2022): 5190, 10.3390/cancers14215190. 36358608 PMC9653806

[31] M. Manuel , O. Tredan , T. Bachelot , et al., “Lymphopenia Combined With Low TCR Diversity (divpenia) Predicts Poor Overall Survival in Metastatic Breast Cancer Patients,” Oncoimmunology 1 (2012): 432–440, 10.4161/onci.19545. 22754761 PMC3382902

[32] B. P. Venkatesulu , S. Mallick , S. H. Lin , and S. Krishnan , “A Systematic Review of the Influence of Radiation‐Induced Lymphopenia on Survival Outcomes in Solid Tumors,” Critical Reviews in Oncology/Hematology 123 (2018): 42–51, 10.1016/j.critrevonc.2018.01.003. 29482778

[33] H. Zeng , W. Zhang , Y. Gong , and C. Xie , “Radiotherapy Activates Autophagy to Increase CD8+ T Cell Infiltration by Modulating Major Histocompatibility Complex Class‐I Expression in Non‐Small Cell Lung Cancer,” Journal of International Medical Research 47, no. 8 (2019): 3818–3830, 10.1177/0300060519855595. 31187666 PMC6726798

[34] J. H. Heinzerling , O. V. Pen , M. Robinson , et al., “Full Dose SBRT in Combination With Mediastinal Chemoradiation for Locally Advanced, Non‐Small Cell Lung Cancer: A Practical Guide for Planning, Dosimetric Results From a Phase 2 Study, and a Treatment Planning Guide for the Phase 3 NRG Oncology LU‐008 Trial,” Practical Radiation Oncology 13, no. 6 (2023): 531–539, 10.1016/j.prro.2023.04.014. 37406774

[35] T. C. Wu , E. Luterstein , B. K. Neilsen , et al., “Accelerated Hypofractionated Chemoradiation Followed by Stereotactic Ablative Radiotherapy Boost for Locally Advanced, Unresectable Non‐small Cell Lung Cancer: a Nonrandomized Controlled Trial,” JAMA Oncology 10, no. 3 (2024): 352–359, 10.1001/jamaoncol.2023.6033. 38206614 PMC10784998

[36] S. E. Schild , H. H. Pang , W. Fan , et al., “Exploring Radiotherapy Targeting Strategy and Dose: a Pooled Analysis of Cooperative Group Trials of Combined Modality Therapy for Stage III NSCLC,” Journal of Thoracic Oncology 13, no. 8 (2018): 1171–1182, 10.1016/j.jtho.2018.04.011. 29689435

[37] T. Abe , M. Iino , S. Saito , et al., “Feasibility of Intensity Modulated Radiotherapy With Involved Field Radiotherapy for Japanese Patients With Locally Advanced Non‐Small Cell Lung Cancer,” Journal of Radiation Research 62 (2021): 894–900, 10.1093/jrr/rrab063. 34260719 PMC8438249

[38] E. Topkan , Y. Ozdemir , O. C. Guler , et al., “Comparison of Involved Field Radiotherapy Versus Elective Nodal Irradiation in Stage IIIB/C Non‐Small‐Cell Lung Carcinoma Patients Treated With Concurrent Chemoradiotherapy: a Propensity Score Matching Study,” Journal of Oncology 2020 (2020): 7083149, 10.1155/2020/7083149. 32952557 PMC7487114

[39] C. Kong , X. Zhu , M. Shi , et al., “Survival and Toxicity of Hypofractionated Intensity Modulated Radiation Therapy in 4 Gy Fractions for Unresectable Stage III Non‐Small Cell Lung Cancer,” International Journal of Radiation Oncology, Biology, Physics 107, no. 4 (2020): 710–719, 10.1016/j.ijrobp.2020.03.038. 32275994

